# Dementia risk and thalamic nuclei volumetry in healthy midlife adults: the PREVENT Dementia study

**DOI:** 10.1093/braincomms/fcae046

**Published:** 2024-02-15

**Authors:** Sita N Shah, Maria-Eleni Dounavi, Paresh A Malhotra, Brian Lawlor, Lorina Naci, Ivan Koychev, Craig W Ritchie, Karen Ritchie, John T O’Brien

**Affiliations:** Department of Psychiatry, University of Cambridge School of Clinical Medicine, Cambridge CB2 0QQ, UK; Department of Psychiatry, University of Cambridge School of Clinical Medicine, Cambridge CB2 0QQ, UK; Department of Brain Sciences, Imperial College London, London W12 0NN, UK; UK Dementia Research Institute Care Research and Technology Centre, Imperial College London and the University of Surrey, London SW7 2AZ, UK; Trinity College Institute of Neuroscience, School of Psychology, Trinity College Dublin, Dublin D02 PX31, Ireland; Global Brain Health Institute, Trinity College Dublin, Dublin D02 X9W9, Ireland; Trinity College Institute of Neuroscience, School of Psychology, Trinity College Dublin, Dublin D02 PX31, Ireland; Global Brain Health Institute, Trinity College Dublin, Dublin D02 X9W9, Ireland; Department of Psychiatry, University of Oxford, Oxford OX3 7JX, UK; Centre for Dementia Prevention, Centre for Clinical Brain Sciences, University of Edinburgh, Edinburgh EH4 2XU, UK; Institute de Neurosciences de Montpellier, INSERM, Montpellier 34093, France; Department of Psychiatry, University of Cambridge School of Clinical Medicine, Cambridge CB2 0QQ, UK

**Keywords:** preclinical dementia, volumetric analysis, thalamic segmentation, neuroimaging biomarkers

## Abstract

A reduction in the volume of the thalamus and its nuclei has been reported in Alzheimer’s disease, mild cognitive impairment and asymptomatic individuals with risk factors for early-onset Alzheimer’s disease. Some studies have reported thalamic atrophy to occur prior to hippocampal atrophy, suggesting thalamic pathology may be an early sign of cognitive decline. We aimed to investigate volumetric differences in thalamic nuclei in middle-aged, cognitively unimpaired people with respect to dementia family history and apolipoprotein ε4 allele carriership and the relationship with cognition. Seven hundred participants aged 40–59 years were recruited into the PREVENT Dementia study. Individuals were stratified according to dementia risk (approximately half with and without parental dementia history). The subnuclei of the thalamus of 645 participants were segmented on T_1_-weighted 3 T MRI scans using FreeSurfer 7.1.0. Thalamic nuclei were grouped into six regions: (i) anterior, (ii) lateral, (iii) ventral, (iv) intralaminar, (v) medial and (vi) posterior. Cognitive performance was evaluated using the computerized assessment of the information-processing battery. Robust linear regression was used to analyse differences in thalamic nuclei volumes and their association with cognitive performance, with age, sex, total intracranial volume and years of education as covariates and false discovery rate correction for multiple comparisons. We did not find significant volumetric differences in the thalamus or its subregions, which survived false discovery rate correction, with respect to first-degree family history of dementia or apolipoprotein ε4 allele status. Greater age was associated with smaller volumes of thalamic subregions, except for the medial thalamus, but only in those without a dementia family history. A larger volume of the mediodorsal medial nucleus (*P*_false discovery rate_ = 0.019) was associated with a faster processing speed in those without a dementia family history. Larger volumes of the thalamus (*P* = 0.016) and posterior thalamus (*P*_false discovery rate_ = 0.022) were associated with significantly worse performance in the immediate recall test in apolipoprotein ε4 allele carriers. We did not find significant volumetric differences in thalamic subregions in relation to dementia risk but did identify an interaction between dementia family history and age. Larger medial thalamic nuclei may exert a protective effect on cognitive performance in individuals without a dementia family history but have little effect on those with a dementia family history. Larger volumes of posterior thalamic nuclei were associated with worse recall in apolipoprotein ε4 carriers. Our results could represent initial dysregulation in the disease process; further study is needed with functional imaging and longitudinal analysis.

## Teaser

Shah *et al*. investigated thalamic nuclei volumetry and the effect on cognition in middle-aged individuals. Larger medial thalamic nuclei were associated with faster processing speeds in individuals without a dementia family history, while larger volumes of several posterior thalamic nuclei were associated with worse immediate recall ability in apolipoprotein ε4 carriers.

## Graphical Abstract

**Graphical Abstract:**
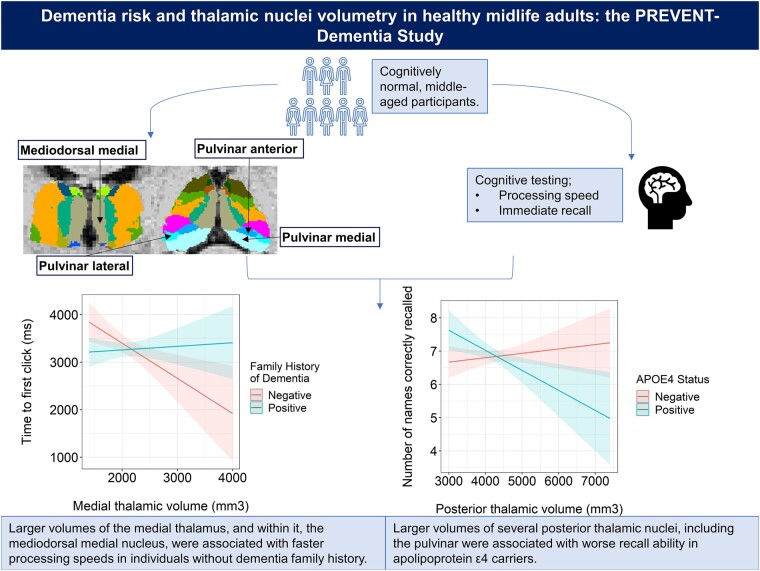

## Introduction

Alzheimer’s disease is the most common cause of dementia, characterized by cognitive decline associated with the presence of amyloid beta plaques and neurofibrillary tangles.^1^ Some of the earliest symptoms that present during the prodromal stages include disturbances in mood, sleep and motivation.^2^ Cognitive domains affected include episodic memory, visuospatial awareness, executive function, attention, speech and language.^2^

Inherited autosomal dominant genetic mutations in the amyloid precursor protein (APP) and presenilin 1 (PSEN1) and 2 (PSEN2) genes cause early-onset Alzheimer’s disease, which presents before the age of 65 years.^3^ Mutations in these genes result in the excess production and accumulation of amyloid beta 42 (Aβ_42_).^3^ Individuals with these mutations display a strong family history for early-onset Alzheimer’s disease.^3^ Most cases of sporadic Alzheimer’s disease are of late onset, typically presenting in individuals over 65 years of age and associated with several genetic risk factors, particularly possession of the apolipoprotein ε4 (APOE4) allele.^4^ Homozygote APOE4 carriers have a greater risk of developing Alzheimer’s dementia than heterozygotes.^4^ APOE4 carriers exhibit greater medial temporal lobe atrophy, tau accumulation and cognitive dysfunction compared to non-carriers and are more likely to experience mild cognitive impairment (MCI) prior to overt dementia.^5-8^

Much of the focus on brain changes at the earliest stages of disease have been on the hippocampus and medial temporal lobe.^9^ However, other structures, like the thalamus and posterior brain regions, have shown early pathological changes in the Alzheimer’s disease process.^10-12^ A reduction in the volume of these areas has been shown to correlate with poorer cognition in people with Alzheimer’s dementia and MCI.^12-15^ Volume loss in the hippocampus and the thalamus appears to precede atrophy of other structures such as the amygdala, where a volume reduction is only seen in people with Alzheimer’s dementia.^16^

The thalamus comprises of 25 nuclei in each brain hemisphere (Table 1).^17^ Multiple studies have shown changes in the thalamic nuclei in Alzheimer’s dementia.^18,19^ At autopsy, amyloid plaques have been found in almost all thalamic nuclei in Alzheimer’s dementia, especially the anteroventral nucleus.^20^ Neurofibrillary tangles have been identified in the anterior and intralaminar thalamic subregions, and the laterodorsal and anteroventral nuclei.^20^ Neuronal loss and neurofibrillary tangles have also been found in the anterodorsal, dorsomedial and pulvinar nuclei.^21-23^

**Table 1:** Thalamic nuclei and their groupings as suggested by Iglesias *et al*.^17^

Group	Nuclei
Anterior	Anteroventral (AV)
Lateral	Laterodorsal (LD)
	Lateral posterior (LP)
Ventral	Ventral anterior (VA)
	Ventral anterior magnocellular (VAmc)
	Ventral lateral anterior (VLa)
	Ventral lateral posterior (VLp)
	Ventral posterolateral (VPL)
	Ventromedial (VM)
Intralaminar	Central medial (CEM)
	Central lateral (CL)
	Paracentral (Pc)
	Centromedian (CM)
	Parafascicular (Pf)
Medial	Paratenial (Pt)
	Reuniens (medial ventral) (MVre)
	Mediodorsal medial magnocellular (MDm)
	Mediodorsal lateral parvocellular (MDl)
Posterior	Lateral geniculate (LGN)
	Medial geniculate (MGN)
	Limitans (suprageniculate) (LSG)
	Pulvinar anterior (PuA)
	Pulvinar medial (PuM)
	Pulvinar lateral (PuL)
	Pulvinar inferior (PuI)
Others	Reticular

Only a few studies have investigated volumetric changes in the thalamic nuclei in individuals with early- and late-onset Alzheimer’s disease and MCI. Asymptomatic and symptomatic PSEN1 and APP mutation carriers have been found to have reduced thalamic volumes compared to healthy controls, while those with dementia exhibited a reduction in temporal and parietal volumes.^24^ A reduction in the volumes of the mediodorsal, pulvinar and medial geniculate (MGN) nuclei has been identified in early MCI.^25^ The same nuclei, with the addition of the anteroventral and centromedian nuclei, are significantly smaller in individuals with late MCI and Alzheimer’s dementia compared to healthy controls.^25^ Van de Mortel *et al.*^26^ found the volume of the ventrolateral thalamus was reduced in those with early MCI compared to those with normal cognition. Hippocampal and temporal atrophy only became evident in late MCI and Alzheimer’s dementia, suggesting that thalamic pathology may be one of the first signs of cognitive decline.^26^ Low *et al.*^27^ have identified significantly smaller anterior and posterior thalamic subnuclei volumes and greater leftward ventral thalamic atrophy in Alzheimer’s dementia compared to MCI and healthy controls.

The thalamus plays a key role in cognition, especially in recall and processing speed.^28,29^ Deficits in episodic memory and executive function have been noted, with executive dysfunction being linked to damage in the medial thalamus.^30,31^ Individuals exhibit difficulties with cognitive flexibility following a thalamic stroke.^31^ They also display difficulties with recognition and a greater response time, which is associated with volume loss of the mediodorsal lateral nuclei.^32^ Other case reports of thalamic strokes involving the mediodorsal medial (MDm) and lateral territory have reported greater problems with recall rather than recognition.^33^ Processing speed has been shown to be related to the thalamus in individuals with multiple sclerosis.^34,35^ Atrophy of the anterior and superior left thalamus has been associated with slower processing speeds.^29^

Collectively, there is evidence suggesting the thalamus is affected early in the pathological course of Alzheimer’s disease and may even predate hippocampal changes.^26^ Thalamic pathology may be implicated in the initial cognitive changes during the prodromal stages of Alzheimer’s disease. A better understanding of the sequence of structural brain changes and cognitive performance can identify biomarkers to prevent dementia in those at risk.

We aimed to investigate differences in the volumes of the thalamus and its nuclei and the effect on cognitive performance with respect to dementia family history (FHD) and APOE4 carriership in a middle-aged, cognitively normal cohort, without signs of global atrophy.^36^ We hypothesized the volume of the thalamus would be reduced in APOE4 carriers compared to non-carriers and also reduced in individuals with FHD compared to those without. We further hypothesized that volumetric loss would be more prominent in the anterior and medial thalamic nuclei and would be related to recall ability.

## Materials and methods

### Participants

Seven hundred cognitively healthy (absence of dementia or other neurological or major psychiatric conditions) participants aged between 40 and 59 years were recruited to the PREVENT Dementia study, which includes four sites in the UK (Oxford, Cambridge, Edinburgh and West London) and one in Ireland (Dublin). The study protocol has been described extensively before.^37^ A variety of recruitment strategies were used with the aim of purposefully recruiting a cohort where approximately half the participants had a parental FHD and half did not. Strategies included approaching children of individuals with dementia using the dementia register database from the West London Mental Health National Health Service (NHS) Trust, using other local site volunteer registers, word of mouth, the Join Dementia Research website and PREVENT Dementia website (https://www.joindementiaresearch.nihr.ac.uk/ and https://preventdementia.co.uk/). All subjects provided written informed consent, and the study was approved by the London-Camberwell St Giles NHS Ethics Committee (REC reference; 12/LO/1023), which acts in accordance with the Declaration of Helsinki of 1975 (and as revised in 1983). Participants were stratified according to FHD, which was defined as having one or both parents with dementia.

All participants underwent cognitive testing and MRI imaging at one of the five sites. Participants underwent APOE4 genotyping using QuantStudio12K. Those without FHD or APOE4 alleles were control subjects for each respective analysis.

### Cognitive analysis

Participants underwent cognitive testing using the computerized assessment of information (COGNITO) battery.^38^ This computerized cognitive battery tests a wide range of cognitive domains, such as attention, language, visuospatial awareness and memory, and has demonstrated good test–retest reliability.^38^ The following measures and tests were used for the cognitive analyses:

- Number of names correctly recalled in the immediate recall test (test of recall ability). Participants were asked to list the nine names that had been read aloud to them immediately after acquisition.
- Number of names correctly recalled in the delayed recall test (test of recall ability). Participants had to recite the names they had previously learned after completing other tests.
- Number of elements correctly recalled in the descriptive recall test (test of recall ability). Subjects had to recall 27 visual descriptive words or phrases they had been told as part of a short descriptive excerpt.
- Number of elements correctly recalled in the narrative recall test (test of recall ability). Participants had to recall 27 elements that had been told to them in a logical sequence as part of a story.
- Time to first click in the visual and auditory attention test (test of working memory), which was used as a marker of processing speed. Participants were shown a shape and had to click on the identical shape while counting the sounds that were being played.
- Number of correct trials in the Stroop test (test of cognitive flexibility). Participants had to match the name of a colour to the button containing the name of the colour, a picture of the colour to the name of the colour and the name of the colour written in that colour to the colour name button.
- These tests were selected as they have previously been shown to be sensitive to thalamic dysfunction.^28,29,31-35,39^

### Image analysis

As part of the PREVENT Dementia MRI protocol, a T_1_-weighted magnetization-prepared rapid gradient echo sequence was acquired on 3 T Siemens (Siemens Healthcare, Erlangen, Germany) scanners with models varying depending on the scanning site (Prisma, Verio, Prisma fit and Skyra).

**Figure 1:**
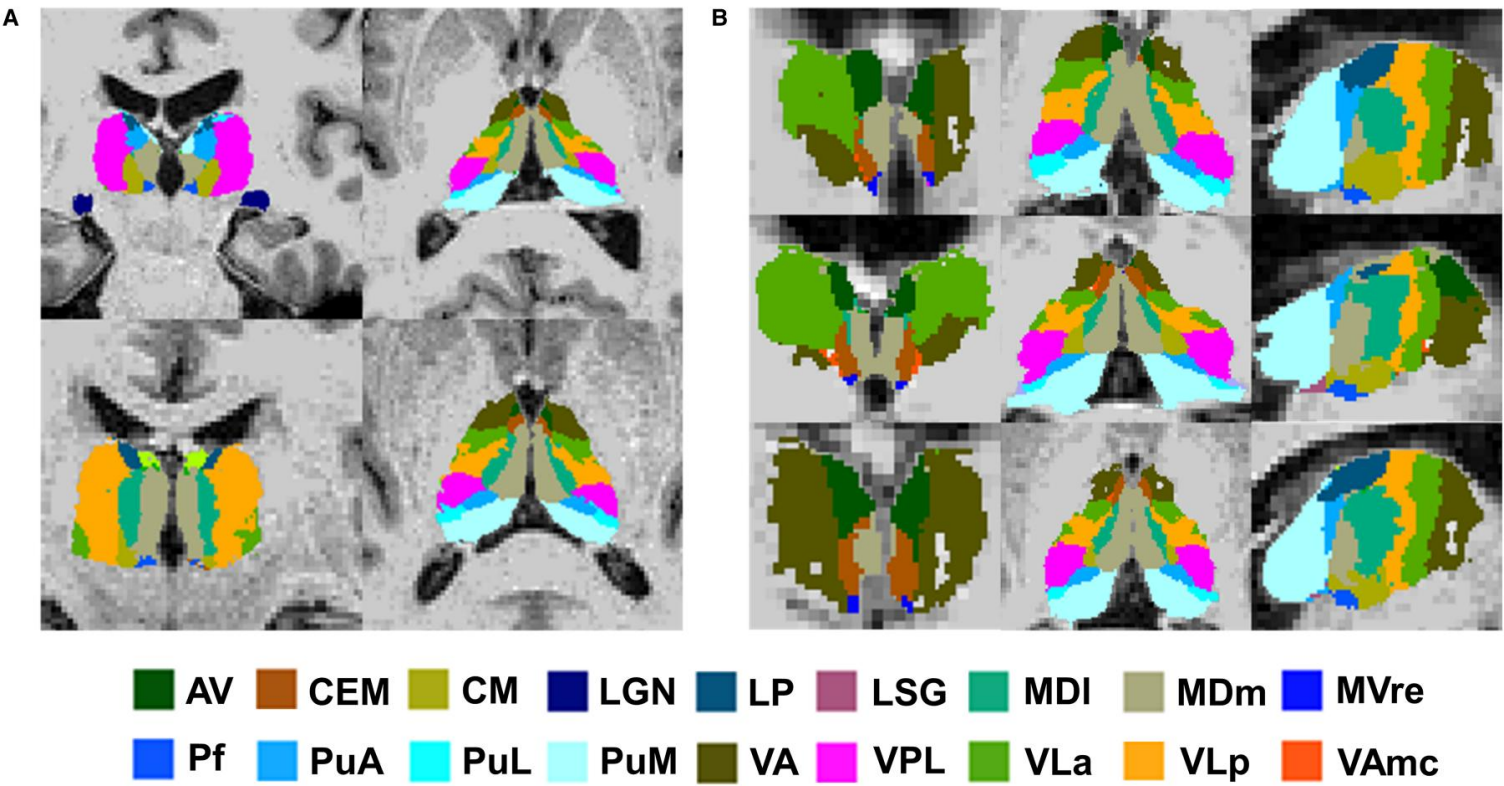
**Examples of the thalamic FreeSurfer segmentation output and the nuclei shown.** (**A**) Coronal and axial slices from two subjects displaying good thalamic segmentation. (**B**) Coronal, axial and sagittal slices from three subjects displaying failed segmentation. AV, anteroventral; CEM, central medial; CM, centromedian; LGN, lateral geniculate; LP, lateral posterior; LSG, limitans (suprageniculate); MDI, mediodorsal lateral; MDm, mediodorsal medial; MVre, reuniens (medial ventral); Pf, parafascicular; PuA, pulvinar anterior; PuL, pulvinar lateral; PuM, pulvinar medial; VA, ventral anterior; VPL, ventral posterolateral; VLa, ventral lateral anterior; VLp, ventral lateral posterior; VAmc, ventral anterior magnocellular.

Participants were excluded if they did not have an MRI scan (*n* = 55) available. The remaining 645 T_1_-weighted MRI scans were analysed using FreeSurfer 7.1.0 software and in particular the recon-all pipeline. Subsequently, a thalamic nuclei segmentation module, which is part of the FreeSurfer software, was applied (https://freesurfer.net/fswiki/ThalamicNuclei) to segment the thalamic nuclei. This software has been validated to segment thalamic nuclei and is based on a probabilistic map determined from post-mortem histology samples and *ex vivo* MRI brain scans.^17^ This is a validated atlas and demonstrates good test–retest reliability.^17^ It can identify 25 nuclei in each thalamic hemisphere. These nuclei were grouped into the following regions: anterior, lateral, ventral, intralaminar, medial and posterior, as suggested by Iglesias *et al.*^17^ and shown in Table 1. Following segmentation, all scans underwent quality control, which was based on visual inspection of the scans. Scans were excluded from analysis if they included incidental findings, pathological lesions, or if the segmentation process had failed. This involved scans where the segmentation was missing several contiguous voxels or contained a lesion, as seen in Fig. 1. Fifteen scans were excluded due to pathological findings, and a further 15 scans because of failed segmentation, leaving 615 included for further analysis.

Thalamic volumes underwent ComBat harmonization to account for differences in the scanning equipment across the five sites. ComBat harmonization is used to account for the variability attributed to different sites and/or scanners.^40^ Age, sex, years of education, APOE4 and FHD were used as modulating variables. Four subjects were excluded from analysis looking at the effect of FHD due to incomplete information about their FHD. Four different subjects had incomplete information about APOE4 status and were excluded for analyses involving APOE4 status. Six hundred and eleven participants were included for analyses involving FHD, and 611 subjects were included for analysis involving APOE4 status.

### Statistical analysis

Statistical analyses were carried out using R Statistical Software (version 4.1.0). A Shapiro–Wilk test was used to assess normality. Robust linear regression was used with age, sex, years of education and total intracranial volume as covariates. Initial analyses were confined to the thalamus and its six subregions, as suggested by Iglesias *et al.*,^17^ to identify any subtle changes in this young cohort. A false discovery rate (FDR) method was applied to correct for multiple comparisons. A *P* < 0.05 was considered statistically significant. For any regions with a significant *P*_FDR_-value, further analysis investigating the nuclei within that region, as shown in Table 1, was undertaken with FDR correction.

Chi-squared and Wilcoxon rank-sum tests were used to check for any statistical differences in baseline characteristics between the two groups. Volumetric differences of the thalamus and its subregions were compared with respect to FHD and APOE4 status. An interaction analysis was carried out to evaluate the effect of volumes of the regions of interest (ROIs) and FHD or APOE4 on cognitive performance. The effect of interactions between age and FHD and age and APOE4 on volumes of the ROI was investigated. Spearman’s correlation was used to determine the directionality of significant interactions.

## Results

The baseline characteristics for those included are summarized in Table 2. There were significant differences in the number of APOE4 carriers between FHD-positive and FHD-negative groups (*P* = 0.002) and the mean age (*P* = 0.037) and number of individuals with FHD (*P* = 0.002) between APOE4 carriers and non-carriers.

**Table 2:** Summary of the demographics of the study participants

Characteristic	Total, *n* = 615	FHD positive, *n* = 325	FHD negative, *n* = 286	APOE4 carrier, *n* = 235	APOE4 non-carrier, *n* = 376
Mean age ± SD (years)	51.1 ± 5.4	51.6 ± 4.9	50.6 ± 6.0	50.6 ± 5.5	51.5 ± 5.4
Mean years of education ± SD	16.7 ± 3.4	16.5 ± 3.2	16.9 ± 3.7	16.9 ± 3.6	16.6 ± 3.3
FHD	Positive: 52.8%; negative: 46.5%	N/A	N/A	Positive: 60.4%; negative: 38.7%	Positive: 47.6%; negative: 51.9%
APOE4 status	Carrier: 38.2%; non-carrier: 61.2%	Carrier: 43.7%; non-carrier: 55.1%	Carrier: 31.8%; non-carrier: 68.2%	N/A	N/A
Sex	Male: 38.2%; female: 61.8%	Male: 36.0%; female: 64.0%	Male: 40.9%; female: 59.1%	Male: 37.0%; female: 63.0%	Male: 39.1%; female: 60.9%

SD, standard deviation; APOE4, apolipoprotein ε4; FHD, dementia family history (parental); N/A, not applicable.

### Volumetric differences with respect to FHD

**Figure 2:**
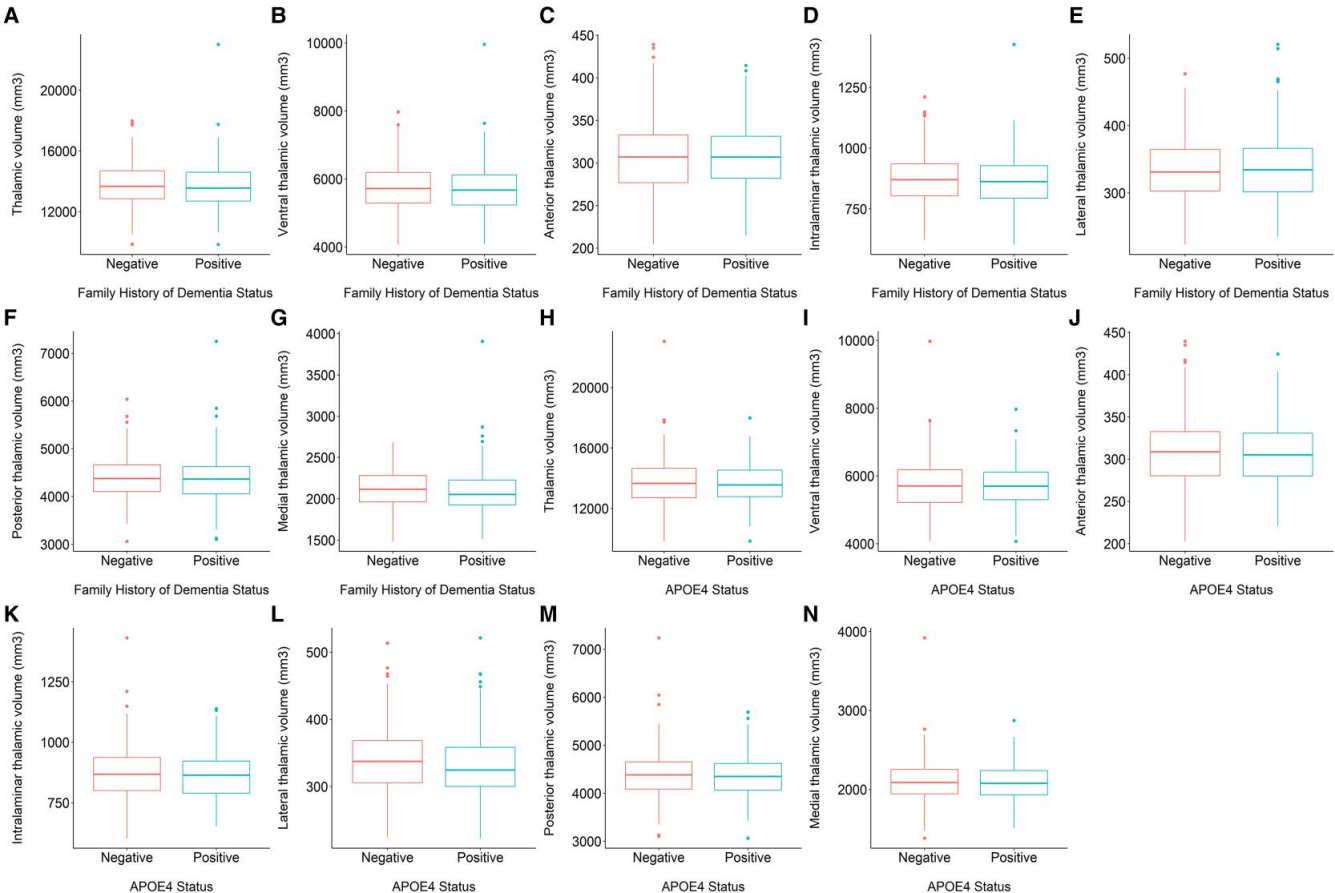
**The effect of dementia risk on thalamic volumes.** Boxplots showing the differences in the raw, unadjusted volumes of the (**A**) thalamus (robust linear regression, *t* = −0.680, *P* = 0.497); (**B**) ventral thalamus (robust linear regression, *t* = −0.845, *P*_FDR_ = 0.683); (**C**) anterior thalamus (robust linear regression, *t* = 0.203, *P*_FDR_ = 0.839); (**D**) intralaminar thalamus (robust linear regression, *t* = −0.748, *P*_FDR_ = 0.683); (**E**) lateral thalamus (robust linear regression, *t* = 0.901, *P*_FDR_ = 0.683); (**F**) posterior thalamus (robust linear regression, *t* = 0.432, *P*_FDR_ = 0.799); and (**G**) medial thalamus (robust linear regression, *t* = −2.417, *P*_FDR_ = 0.095) with respect to FHD. Boxplots showing the differences in the raw, unadjusted volumes of the (**H**) thalamus (robust linear regression, *t* = 0.105, *P* = 0.917); (**I**) ventral thalamus (robust linear regression, *t* = 0.275, *P*_FDR_ = 0.994); (**J**) anterior thalamus (robust linear regression, *t* = 0.008, *P*_FDR_ = 0.994); (**K**) intralaminar thalamus (robust linear regression, *t* = −0.781, *P*_FDR_ = 0.994); (**L**) lateral thalamus (robust linear regression, *t* = −2.058, *P*_FDR_ = 0.240); (**M**) posterior thalamus (robust linear regression, *t* = 0.025, *P*_FDR_ = 0.994); and (**N**) medial thalamus (robust linear regression, *t* = −0.519, *P*_FDR_ = 0.994) with respect to APOE4 status.

We did not find a significant difference in the volume of the whole thalamus when comparing FHD-positive and FHD-negative groups (*t* = −0.68, *P* = 0.497). However, individuals with a positive FHD had a trend towards a smaller medial thalamus compared to FHD-negative group (*t* = −2.417, *P* = 0.016, *P*_FDR_ = 0.095, *β* = −0.080), as shown in Fig. 2.

### Volumetric differences with respect to APOE4 status

We did not find a difference in the volume of the thalamus with respect to APOE4 status (*t* = 0.105, *P* = 0.917). We also did not find any volumetric differences of the thalamic subregions with respect to APOE4 that survived FDR correction.

### Interaction between age and Alzheimer’s disease risk factors in predicting thalamic volumes

**Figure 3:**
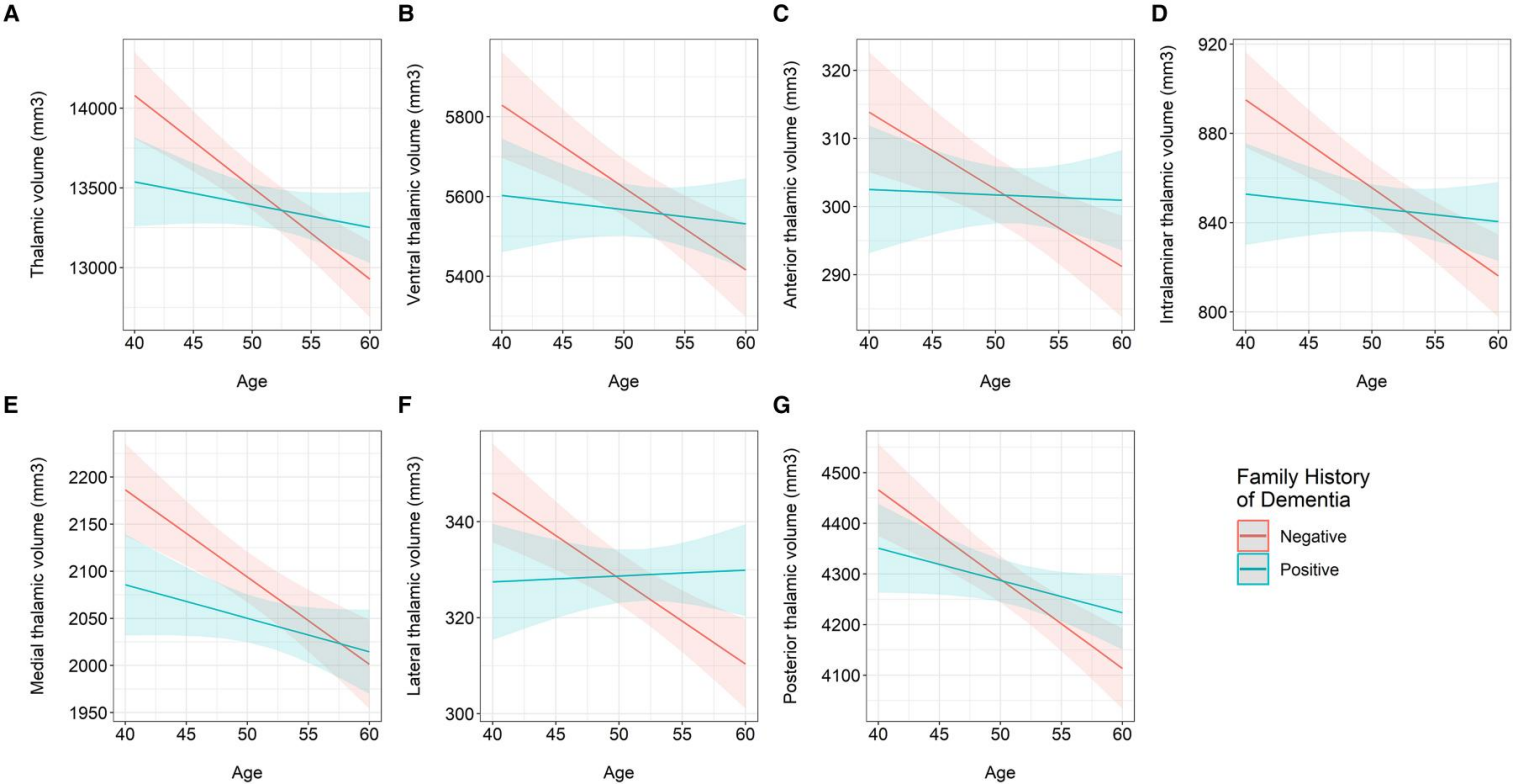
**Interaction between age and FHD on thalamic volumes.** Marginal effects plots showing the interaction between age and FHD in predicting changes in the volumes of the (**A**) thalamus (robust linear regression, *t* = 2.828, *P* = 0.005); (**B**) ventral thalamus (robust linear regression, *t* = 2.226, *P*_FDR_ = 0.040); (**C**) anterior thalamus (robust linear regression, *t* = 2.115, *P*_FDR_ = 0.042); (**D**) intralaminar thalamus (robust linear regression, *t* = 2.754, *P*_FDR_ = 0.018); (**E**) medial thalamus (robust linear regression, *t* = 1.922, *P*_FDR_ = 0.055); (**F**) lateral thalamus (robust linear regression, *t* = 3.025, *P*_FDR_ = 0.016); and (**G**) posterior thalamus (robust linear regression, *t* = 2.231, *P*_FDR_ = 0.040).

There were significant interactions between age and FHD status in predicting volumes of the thalamus and its subregions except the medial thalamus, as shown in Fig. 3 and Supplementary Fig. 1 (thalamus, *t* = 2.828, *P* = 0.005; anterior, *t* = 2.115, *P* = 0.035, *P*_FDR_ = 0.042; ventral, *t* = 2.226, *P* = 0.026, *P*_FDR_ = 0.04; intralaminar, *t* = 2.754, *P* = 0.006, *P*_FDR_ = 0.018; lateral, *t* = 3.025, *P* = 0.003, *P*_FDR_ = 0.016; medial, *t* = 1.922, *P* = 0.055, *P*_FDR_ = 0.055; posterior, *t* = 2.231, *P* = 0.026, *P*_FDR_ = 0.04). Greater age was associated with smaller volumes of these ROIs in the FHD-negative group (Supplementary Table 1; thalamus, *ρ* = −0.153, *P* = 0.010; anterior, *ρ* = −0.103, *P* = 0.083; ventral, *ρ* = −0.117, *P* = 0.047; intralaminar, *ρ* = −0.157, *P* = 0.008; lateral, *ρ* = −0.138, *P* = 0.019; posterior, *ρ* = −0.137, *P* = 0.020). We did not find any significant interactions that survived FDR for age and APOE4 in predicting thalamic volumes.

### Associations with cognition with respect to FHD

Cognition was assessed using tasks assessing processing speed, recall and cognitive flexibility. We did not find any significant effects of FHD status alone on performance on any of the cognitive tests that survived FDR correction (immediate recall, *t* = 0.565, *P* = 0.572, *P*_FDR_ = 0.969; delayed recall, *t* = 2.454, *P* = 0.014, *P*_FDR_ = 0.086; descriptive recall, *t* = 2.157, *P* = 0.031, *P*_FDR_ = 0.094; narrative recall, *t* = −0.244, *P* = 0.807, *P*_FDR_ = 0.969; processing speed, *t* = −0.452, *P* = 0.651, *P*_FDR_ = 0.969; Stroop test, *t* = −0.029, *P* = 0.977, *P*_FDR_ = 0.977).

**Figure 4:**
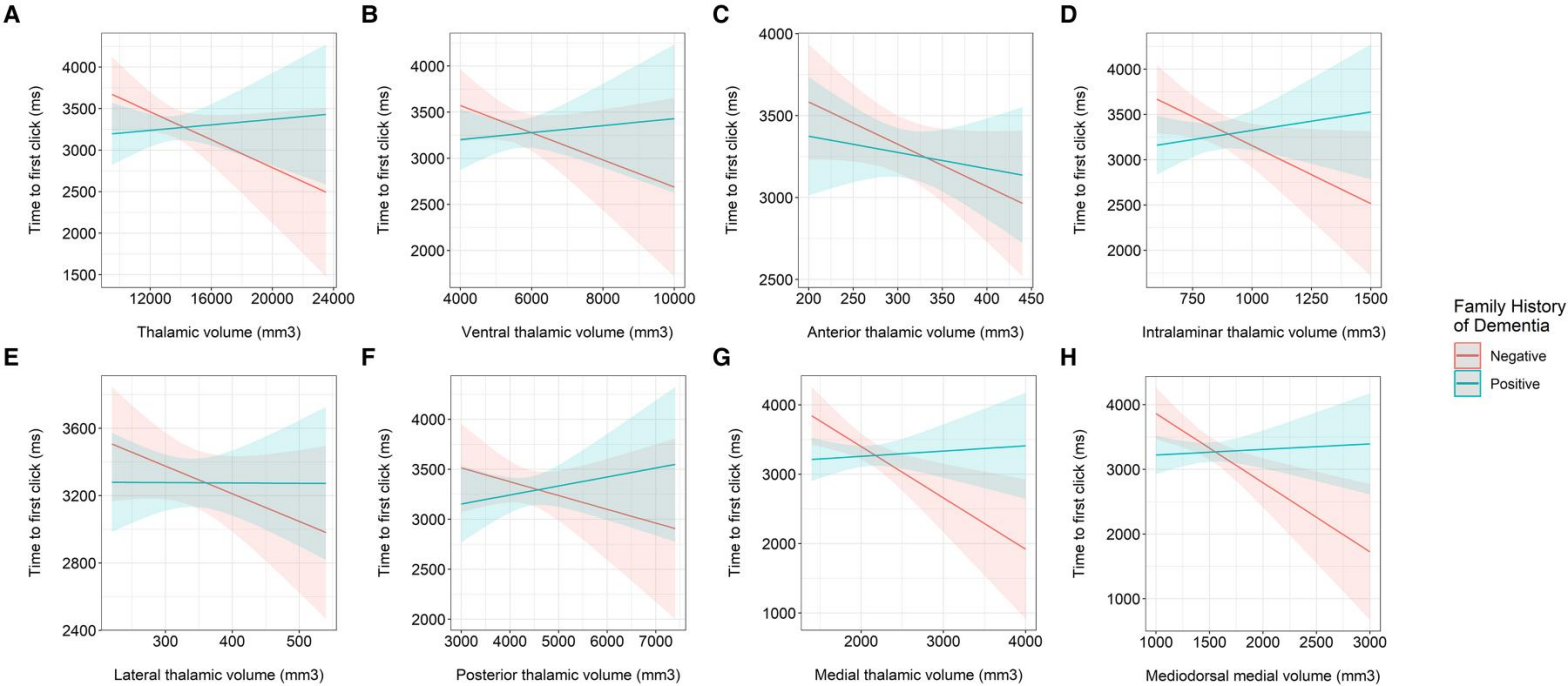
**Interaction between FHD and thalamic volumes in predicting processing speed.** Marginal effects plots showing the interaction between FHD groups and volumes of the (**A**) thalamus (robust linear regression, *t* = 1.864, *P* = 0.063); (**B**) ventral thalamus (robust linear regression, *t* = 1.594, *P*_FDR_ = 0.223); (**C**) anterior thalamus (robust linear regression, *t* = 0.812, *P*_FDR_ = 0.417); (**D**) intralaminar thalamus (robust linear regression, *t* = 2.276, *P*_FDR_ = 0.070), (**E**) lateral thalamus (robust linear regression, *t* = 1.076, *P*_FDR_ = 0.339); (**F**) posterior thalamus (robust linear regression, *t* = 1.356, *P*_FDR_ = 0.263); (**G**) medial thalamus (robust linear regression, *t* = 2.659, *P*_FDR_ = 0.048); and (**H**) MDm (robust linear regression, *t* = 2.831, *P*_FDR_ = 0.019) in predicting processing speed. A shorter time to first click indicates a faster processing speed.

FHD status alone had no impact on the latency of responses in the visual and auditory attention test, as a marker of processing speed. However, there was a significant interaction between FHD and volume of the medial thalamus (*t* = 2.659, *P* = 0.008, *P*_FDR_ = 0.048) in predicting processing speed as displayed in Fig. 4 and Supplementary Fig. 2. Within the medial thalamus, there were significant interactions between the MDm nucleus and FHD (*t* = 2.831, *P* = 0.005, *P*_FDR_ = 0.019) in predicting processing speed. A larger volume of the MDm in those without FHD was associated with shorter time to first click, indicating a faster processing speed (Supplementary Table 2; medial thalamus, *ρ* = −0.255, *P* < 0.001; MDm, *ρ* = −0.260, *P* < 0.001). We did not find any significant interactions between the volumes of the ROI and FHD status on performance on the immediate, delayed, descriptive and narrative recall tests and Stroop test.

### Associations with cognition with respect to APOE4 status

We did not find any significant differences in performance on any of the cognitive tests with respect to APOE4 status alone (immediate recall, *t* = −0.405, *P* = 0.685, *P*_FDR_ = 0.822; delayed recall, *t* = 0.824, *P* = 0.410, *P*_FDR_ = 0.806; descriptive recall, *t* = 1.217, *P* = 0.224, *P*_FDR_ = 0.806; narrative recall, *t* = 0.882, *P* = 0.378, *P*_FDR_ = 0.806; processing speed, *t* = 0.617, *P* = 0.537, *P*_FDR_ = 0.806; Stroop test, *t* = −0.223, *P* = 0.823, *P*_FDR_ = 0.823).

**Figure 5:**
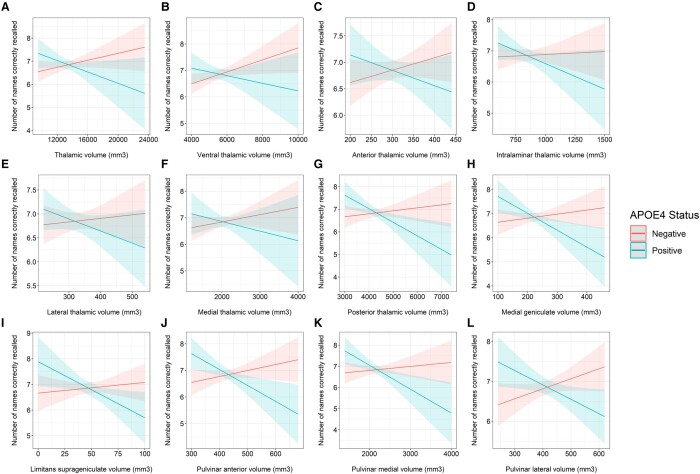
**Interaction between APOE4 and thalamic volumes on immediate recall.** Marginal effects plots showing the interaction between APOE4 groups and volumes of the (**A**) thalamus (robust linear regression, *t* = −2.416, *P* = 0.016); (**B**) ventral thalamus (robust linear regression, *t* = −2.115, *P*_FDR_ = 0.105); (**C**) anterior thalamus (robust linear regression, *t* = −1.758, *P*_FDR_ = 0.143); (**D**) intralaminar thalamus (robust linear regression, *t* = −1.670, *P*_FDR_ = 0.143); (**E**) lateral thalamus (robust linear regression, *t* = −1.389, *P*_FDR_ = 0.198); (**F**) medial thalamus (robust linear regression, *t* = −1.279, *P*_FDR_ = 0.201); (**G**) posterior thalamus (robust linear regression, *t* = −2.914, *P*_FDR_ = 0.022); (**H**) MGN (robust linear regression, *t* = −2.755, *P*_FDR_ = 0.011); (**I**) LSG (robust linear regression, *t* = −2.268, *P*_FDR_ = 0.033); (**J**) PuA (robust linear regression, *t* = −3.256, *P*_FDR_ = 0.008); (**K**) PuM (robust linear regression, *t* = −2.936, *P*_FDR_ = 0.008); and (**L**) PuL (robust linear regression, *t* = −2.930, *P*_FDR_ = 0.008) nuclei in predicting the number of names correctly recalled in the immediate recall test.

There was a significant interaction between the volumes of the thalamus (*t* = −2.416, *P* = 0.016) and posterior thalamus (*t* = −2.914, *P* = 0.004, *P*_FDR_ = 0.022) and APOE4 status in predicting the number of names correctly recalled in the immediate recall test (Fig. 5; Supplementary Fig. 3). Within the posterior region, significant interactions were noted between APOE4 status and the limitans suprageniculate (LSG, *t* = −2.268, *P* = 0.024, *P*_FDR_ = 0.033), MGN (*t* = −2.755, *P* = 0.006, *P*_FDR_ = 0.011), pulvinar anterior (PuA, *t* = −3.256, *P* = 0.001, *P*_FDR_ = 0.008), pulvinar medial (PuM, *t* = −2.936, *P* = 0.003, *P*_FDR_ = 0.008) and the pulvinar lateral (PuL, *t* = −2.930, *P* = 0.004, *P*_FDR_ = 0.008) nuclei. Larger volumes in these regions were associated with worse performance in the immediate recall in APOE4 carriers (Supplementary Table 3; thalamus, *ρ* = −0.180, *P* = 0.006; posterior thalamus, *ρ* = −0.234, *P* < 0.001; LSG, *ρ* = −0.185, *P* = 0.004; MGN, *ρ* = −0.150, *P* = 0.021; PuA, *ρ* = −0.237, *P* < 0.001; PuM, *ρ* = −0.241, *P* < 0.001; PuL, *ρ* = −0.209, *P* = 0.001).

We did not find any significant interactions between the volumes of the ROI and APOE4 status in predicting performance in the delayed, descriptive and narrative recall tests, processing speed or Stroop test.

## Discussion

We previously reported that we have not identified any structural changes in whole brain volumes, cortical thickness and hippocampal subfields with respect to FHD and APOE4 status in this cohort.^36^ In the present study, we found that individuals with a positive FHD display a non-significant trend towards a smaller medial thalamus, but not the thalamus itself, compared to those without FHD. We did not find any significant differences in cognitive performance on several tests with respect to FHD status alone. Greater age was associated with smaller volumes of all thalamic regions, except the medial thalamus in individuals without FHD. A larger volume of the MDm nucleus was associated with faster processing speeds in those without FHD. Comparatively, the volumes of these nuclei were not associated with cognitive performance in the FHD-positive group.

We did not find any significant volumetric or cognitive differences with respect to APOE4 alone. However, larger volumes of the thalamus and posterior thalamus, specifically the MGN, LSG, PuA, PuL and PuM, were associated with worse performance on the immediate recall test in APOE4 carriers.

To our knowledge, this is the first study to investigate volumetric changes in the thalamic nuclei in a preclinical cohort at midlife. The volume of the thalamus is reduced in MCI and Alzheimer’s dementia, with some studies reporting thalamic atrophy occurring prior to hippocampal atrophy.^16,26,41-44^ Volumes of several thalamic nuclei such as the mediodorsal, centromedian, pulvinar and medical geniculate are reduced in MCI and Alzheimer’s dementia.^25,45^ Pardilla-Delgado *et al.*^46^ did not find any significant volumetric differences in the thalamus or its subregions but did find smaller volumes of the medial thalamus were associated with amyloid and tau pathology in middle-aged PSEN1 carriers, largely made up of individuals who were cognitively unimpaired and a small number with MCI.^46^ We have identified some non-significant, subtle changes in the thalamic subregions, but it could be that our cohort is too young to observe a significant change yet as it may only be evident in those with higher disease burden.

Greater age was associated with smaller volumes of all thalamic subregions, except for the medial thalamus, in individuals without FHD. It could be that we do not see the same relationship with age in individuals with FHD as the volumes of the thalamus would have already started to decline as part of the disease process.^16,24^ It is well established that thalamic volumes tend to decline with increasing age in cognitively healthy individuals.^47-49^ Thalamic nuclei appear to atrophy at different rates with increasing age in cognitively unimpaired individuals.^50^ The anteroventral, lateral geniculate, MGN, centromedian and pulvinar are some of the nuclei with higher atrophy rates.^50^ Interestingly, Pardilla-Delgado *et al.*^46^ reported a significant negative correlation between age and volumes of the thalamus, and the medial and posterior subregions, but not the other subregions, in non-carriers of the PSEN1 mutation, although this study did not perform correction for multiple comparisons and so results should be considered preliminary. Our results are similar to that from published studies, but it is worth noting that we did not find the same relationship between age and the medial thalamus in individuals without FHD. Our results may indicate the medial thalamus is the last region to atrophy in normal aging. Alternatively, there may be subtle changes occurring in that region with increasing age, which the segmentation algorithm is not sensitive enough to identify.

We found a larger volume of the MDm was associated with a faster processing speed in individuals without FHD. The mediodorsal nucleus is implicated in cognition and cognitive flexibility, primarily due to its link to the prefrontal cortex.^51-53^ Functional MRI (fMRI) has shown increased connectivity in healthy individuals between the hippocampus and several thalamic nuclei such as the mediodorsal, laterodorsal and medioventral at the encoding stage to be associated with delayed recall ability of previously learned associations.^54^ Successful recall tasks have been associated with greater activation of the mediodorsal nucleus and prefrontal cortex during encoding and retrieval, indicating a role for these areas in recall and recognition.^28^ Bilateral MDm lesions in rhesus monkeys impair the acquisition of new memories while leaving previously learned memories intact.^55^ Though we did not find an effect of medial thalamic nuclei volumes and cognition in the FHD-positive group, it could be possible that smaller volumes of these regions may affect cognition later in the disease process.

The effect of APOE4 in midlife is not fully understood. While we did not find any significant volumetric differences, Cacciaglia *et al.*^56^ reported a significant increase in the volume of the right medial thalamus in middle-aged, cognitively unimpaired APOE4 carriers, in a dose-dependent manner, compared to non-carriers. However, some studies have reported no significant differences in cognitive performance with respect to APOE4 alone.^57-59^ Others have found carriers exhibit a greater decline in memory and cognition over time compared to non-carriers.^5,59,60^ Li *et al.*^61^ reported APOE4 carriers had reduced hippocampal functional connectivity to areas such as the thalamus, and episodic memory was positively correlated with hippocampal functional connectivity in carriers, highlighting connectivity changes in components of the Papez circuit evident at midlife.

Rather surprisingly, we found larger volumes of several nuclei in the posterior thalamus were associated with worse performance in the immediate recall test in APOE4 carriers. Cacciaglia *et al.*^58^ demonstrated APOE4 mediates opposite relationships between grey matter volumes and episodic memory and executive function. Scarmeas *et al.*^62^ have reported increased perfusion to regions including the insula, pulvinar and cuneus, accompanied by decreased perfusion to parietal and frontal regions in Alzheimer’s dementia, and this is negatively correlated with Mini Mental State Examination and recall performance.^62^ Our findings could indicate larger volumes of the posterior thalamus and its nuclei may not confer benefit on recall ability. Alternatively, it may represent a change in one area that may only occur in conjunction with alterations with other structures that we have not yet identified.

van de Mortel *et al.*^26^ have found the thalamus is affected early in the disease course, even prior to hippocampal atrophy, suggesting it may be one of the first signs of cognitive decline. The thalamus may be affected early as it forms part of the Papez circuit, which is important for episodic memory.^10^ The thalamus aids in several processes in addition to cognition, such as sleep and hearing, which are affected in the prodromal stages of Alzheimer’s disease, which also indicates an early involvement of the thalamus in the disease process.^2,63,64^ There has been little study of the role of thalamic nuclei in Alzheimer’s disease, and this may be because the precise function of all nuclei is not yet known. As we learn more about the functions of specific nuclei, we will be able to better understand how the thalamus and its nuclei aid in cognition, hearing and sleep. Greater understanding of this could lead to the development of novel neuroimaging biomarkers.

Strengths of our study include our relatively large sample size, as 611 subjects were included in the final analysis. Furthermore, we used a validated segmentation software to segment the thalamus into its nuclei. These scans then underwent a visual quality control inspection to remove any scans with evidence of incidental pathology or failed segmentation to ensure these results did not affect the analysis. To account for differences in scanners used in our five study sites, we performed ComBat harmonization on the volumetric data.

Limitations of our study include the use of 3 T MRI. Our volumetric results did not survive FDR correction, and as such, these results should be considered preliminary, potentially showing some underlying trends, and require replication in other cohorts. A 7 T MRI may be better to identify any subtle volumetric changes. The cross-sectional nature of this study did not permit investigation of the progression of volumetric changes and the effect on cognition over time. In future studies, additional MRI modalities, such as fMRI, may be used to identify functional connectivity changes in the investigated ROI at this stage. Another limitation is that although sizeable, our study may not have been large enough to detect a difference, especially if it is very subtle. Although we could not find a study with published volumes of thalamic nuclei in preclinical stages in individuals at risk of late-onset Alzheimer’s disease, middle-aged PSEN1 mutation carriers have demonstrated a significant difference in the volume of the left thalamus (6962 mm^3^) in presymptomatic PSEN1 mutation carriers compared to healthy controls (7177 mm^3^).^42^ If our population showed similar differences, a much smaller sample size than included here [of 124 in each group (high risk versus low risk)] would have a power of 80%, assuming an alpha value of 0.05 to detect such a difference, though we acknowledge an autosomal dominant group at risk of early-onset Alzheimer’s disease may show larger effects than our sporadic dementia group. A final limitation is that we did not investigate the relationship between volumes of the ROI and markers of Alzheimer’s disease pathology (Aβ_42_ and tau), which are not currently available for the cohort.

In conclusion, we did not find any significant volumetric differences in the thalamic nuclei in relation to FHD or APOE4 allele status, although individuals with a FHD may display a trend towards a smaller volume of the medial thalamus. Greater age was associated with smaller volumes in all thalamic subregions except the medial thalamus in those without FHD. A larger volume of the MDm nucleus was associated with faster processing speeds in individuals without FHD. Contrastingly, larger volumes of several nuclei in the posterior thalamus are associated with worse performance in the immediate recall test in APOE4 carriers. Our findings may indicate an initial dysregulation process, whereby the coupling of thalamic nuclei volumes to cognition changes but neither volumetric nor cognitive deficits are yet apparent in at-risk groups.

## Supplementary Material

fcae046_Supplementary_Data

## Data Availability

The data that support the findings of this study are available upon reasonable request (please see https://preventdementia.co.uk/).
