# Supplementary material for: Dementia risk and thalamic nuclei volumetry in healthy midlife adults: the PREVENT Dementia study
Source: Brain Commun. 2024 Feb 15;6(2):fcae046. doi: 10.1093/braincomms/fcae046 (PMC10914447; doi:10.1093/braincomms/fcae046)
Supplement: fcae046_Supplementary_Data [file fcae046_supplementary_data.pdf]

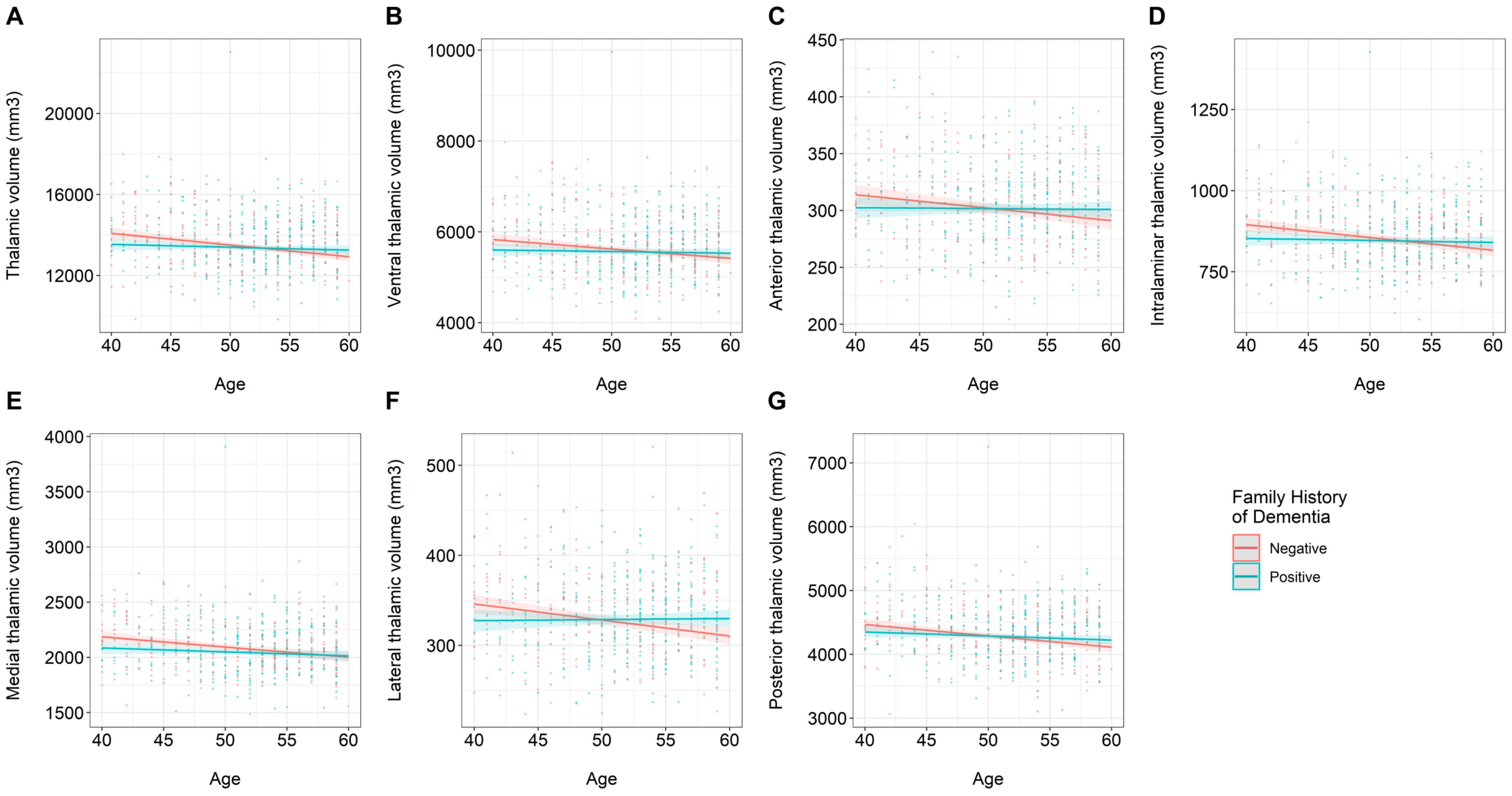

Supplementary figure 1: **Interaction between age and FHD on thalamic volumes. Raw volumes, unadjusted for total intracranial volume, have been plotted.** Marginal effects plots showing the interaction between age and FHD in predicting changes in the volumes of the A) thalamus (robust linear regression, t = 2.828, p = 0.005), B) Ventral thalamus (robust linear regression, t = 2.226, p_{FDR} = 0.040), C) Anterior thalamus (robust linear regression, t = 2.115, p_{FDR} = 0.042), D) Intralaminar thalamus (robust linear regression, t = 2.754, p_{FDR} = 0.018), E) Medial thalamus (robust linear regression, t = 1.922, p_{FDR} = 0.055) , F) Lateral thalamus (robust linear regression, t = 3.025, p_{FDR} = 0.016) and G) Posterior thalamus (robust linear regression, t = 2.231, p_{FDR} = 0.040).

ROI	FHD x age t value	FHD x age p value	FHD x age p_{FDR} value	FHD positive		FHD negative	
				Spearman's ρ	P value	Spearman's ρ	P value
Thalamus	2.828	** 0.005	N/A	-0.011	0.842	-0.153	* 0.010
Anterior	2.115	0.035	* 0.042	0.033	0.553	-0.103	0.083
Ventral	2.226	0.026	* 0.040	0.006	0.919	-0.117	* 0.047
Intralaminar	2.754	0.006	* 0.018	-0.002	0.966	-0.157	** 0.008
Lateral	3.025	0.003	* 0.016	0.046	0.411	-0.138	* 0.019
Medial	1.922	0.055	0.055	N/A	N/A	N/A	N/A
Posterior	2.231	0.026	* 0.040	-0.023	0.681	-0.137	* 0.020

Supplementary table 1: **Statistical summary from the interaction analysis between FHD and age in predicting volumetric changes using robust linear regression.** The Spearman's ρ and the p values are represented for interactions surviving FDR correction. * $p < 0.05$, ** $p < 0.01$. ROI = region of interest, FHD = dementia family history (parental), FDR = false discovery rate.

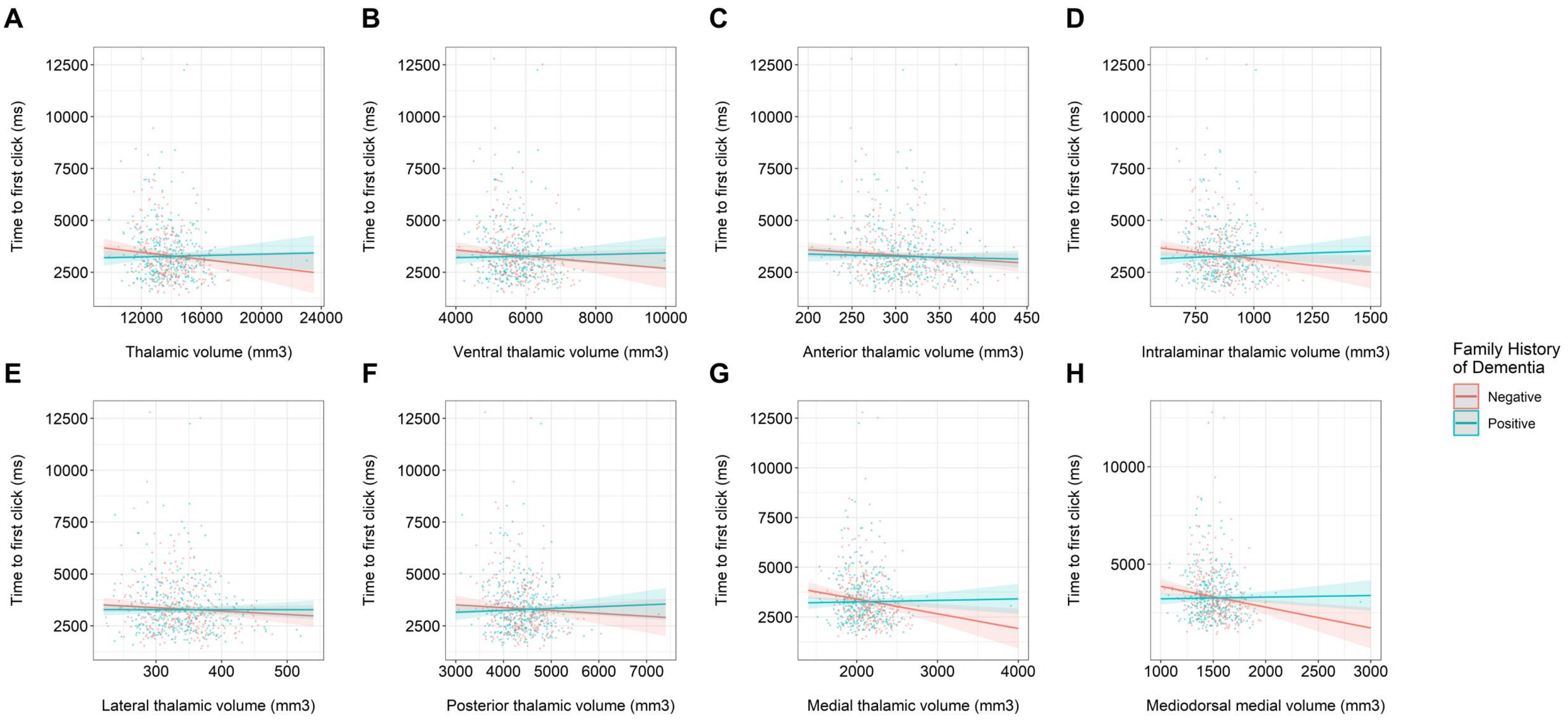

Supplementary figure 2: **Interaction between FHD and thalamic volumes in predicting processing speed. Raw volumes, unadjusted for total intracranial volume, have been plotted.** Marginal effects showing the interaction between FHD groups and volumes of the A) thalamus (robust linear regression, t = 1.864, p = 0.063), B) Ventral thalamus (robust linear regression, t = 1.594, p_{FDR} = 0.223), C) Anterior thalamus (robust linear regression, t = 0.812, p_{FDR} = 0.417), D) Intralaminar thalamus (robust linear regression, t = 2.276, p_{FDR} = 0.070), E) Lateral thalamus (robust linear regression, t = 1.076, p_{FDR} = 0.339), F) Posterior thalamus (robust linear regression, t = 1.356, p_{FDR} = 0.263), G) Medial thalamus (robust linear regression, t = 2.659, p_{FDR} = 0.048) and H) Mediodorsal medial (MDm) (robust linear regression, t = 2.831, p_{FDR} = 0.019) in predicting processing speed. A shorter time to first click indicates a faster processing speed.

ROI	FHD x ROI t value	FHD x ROI p value	FHD x ROI p_{FDR} value	FHD positive		FHD negative	
				Spearman's ρ	P value	Spearman's ρ	P value
Thalamus	1.864	0.063	N/A	N/A	N/A	N/A	N/A
Anterior	0.812	0.417	0.417	N/A	N/A	N/A	N/A
Ventral	1.594	0.112	0.223	N/A	N/A	N/A	N/A
Intralaminar	2.276	0.023	0.070	N/A	N/A	N/A	N/A
Lateral	1.076	0.282	0.339	N/A	N/A	N/A	N/A
Posterior	1.356	0.175	0.263	N/A	N/A	N/A	N/A
Medial	2.659	0.008	* 0.048	-0.074	0.182	-0.255	*** <0.001
MDm	2.831	0.005	* 0.019	-0.080	0.151	-0.260	*** <0.001
Pt	2.021	0.044	0.053	N/A	N/A	N/A	N/A
MVre	1.951	0.052	0.053	N/A	N/A	N/A	N/A
MDl	1.942	0.053	0.053	N/A	N/A	N/A	N/A

Supplementary table 2: **Statistical summary from the interaction analysis between the regions of interest and FHD in predicting processing speeds using robust linear regression**. The Spearman's ρ and the p values are represented for interactions surviving FDR correction. * $p < 0.05$, *** $p < 0.001$. ROI = region of interest, FHD = dementia family history (parental), FDR = false discovery rate, MDm = Mediodorsal medial, Pt = Paratenial, MVre = Reuniens (medial ventral), MDl = Mediodorsal lateral.

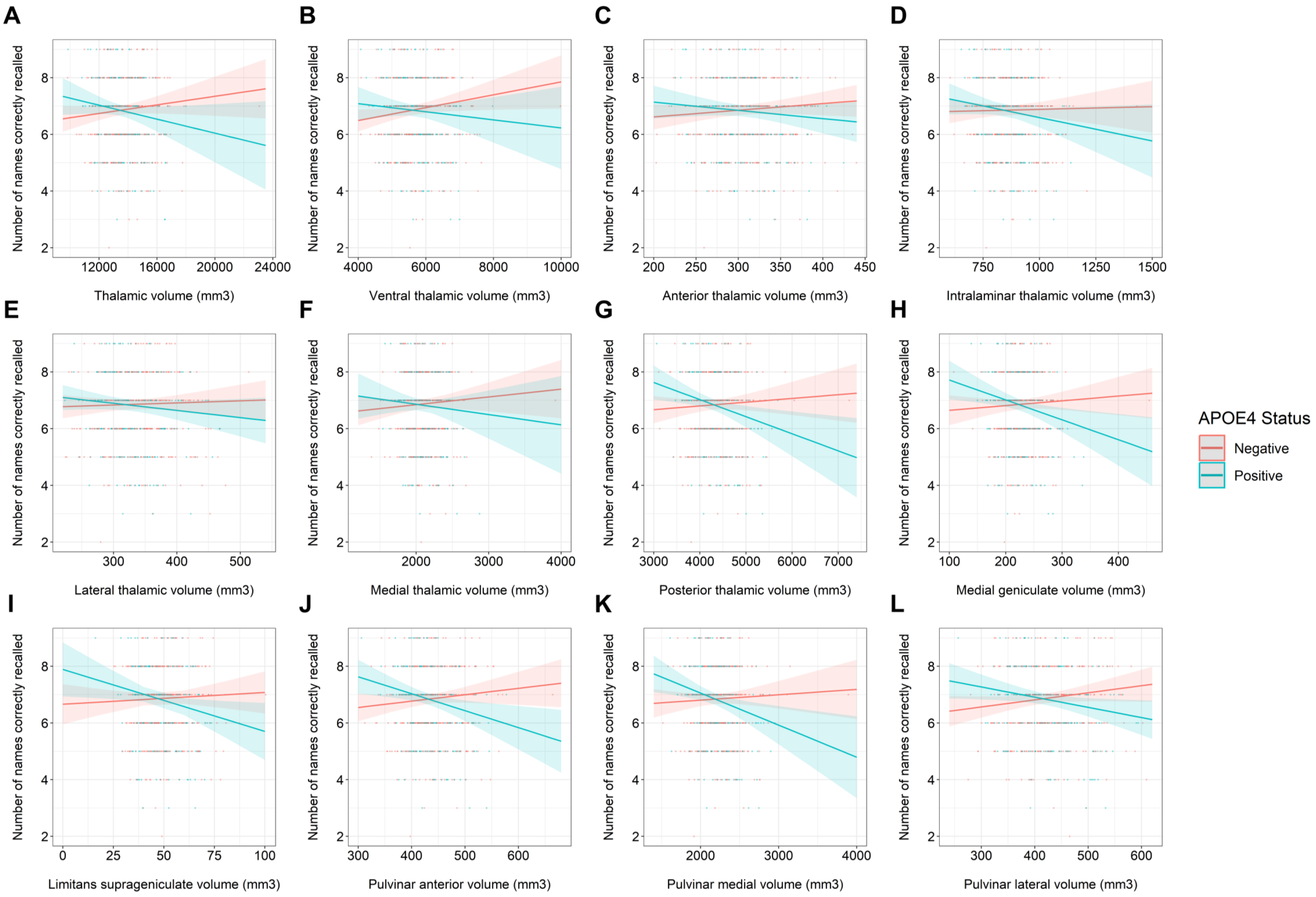

Supplementary figure 3: **Interaction between APOE4 and thalamic volumes on immediate recall. Raw volumes, unadjusted for total intracranial volume, have been plotted.** Marginal effects showing the interaction between APOE4 groups and volumes of the A) thalamus (robust linear regression, $t = -2.416$, $p = 0.016$), B) Ventral thalamus (robust linear regression, $t = -2.115$, $p_{FDR} = 0.105$), C) Anterior thalamus (robust linear regression, $t = -1.758$, $p_{FDR} = 0.143$), D) Intralaminar thalamus (robust linear regression, $t = -1.670$, $p_{FDR} = 0.143$), E) Lateral thalamus (robust linear regression, $t = -1.389$, $p_{FDR} = 0.198$), F) Medial thalamus (robust linear regression, $t = -1.279$, $p_{FDR} = 0.201$), G) Posterior thalamus (robust linear regression, $t = -2.914$, $p_{FDR} = 0.022$), H) Medial geniculate (MGN) (robust linear regression, $t = -2.755$, $p_{FDR} = 0.011$) , I) Limitans suprageniculate (LSG) (robust linear regression, $t = -2.268$, $p_{FDR} = 0.033$), J) Pulvinar anterior (PuA) (robust linear regression, $t = -3.256$, $p_{FDR} = 0.008$), K) Pulvinar medial (PuM) (robust linear regression, $t = -2.936$, $p_{FDR} = 0.008$) and L) Pulvinar lateral (PuL) (robust linear regression, $t = -2.930$, $p_{FDR} = 0.008$) nuclei in predicting the number of names correctly recalled in the immediate recall test.

ROI	APOE4 x ROI t value	APOE4 x ROI p value	APOE4 x ROI p_{FDR} value	APOE4 positive		APOE4 negative	
				Spearman's ρ	P value	Spearman's ρ	P value
Thalamus	-2.416	* 0.016	N/A	-0.180	** 0.006	0.027	0.598
Anterior	-1.758	0.079	0.143	N/A	N/A	N/A	N/A
Ventral	-2.115	0.035	0.105	N/A	N/A	N/A	N/A
Intralaminar	-1.670	0.095	0.143	N/A	N/A	N/A	N/A
Lateral	-1.389	0.165	0.198	N/A	N/A	N/A	N/A
Medial	-1.279	0.201	0.201	N/A	N/A	N/A	N/A
Posterior	-2.914	0.004	* 0.022	-0.234	*** <0.001	0.005	0.927
MGN	-2.755	0.006	* 0.011	-0.150	* 0.021	0.024	0.647
LSG	-2.268	0.024	* 0.033	-0.185	** 0.004	0.024	0.643
PuA	-3.256	0.001	** 0.008	-0.237	*** <0.001	0.035	0.494
PuL	-2.930	0.004	** 0.008	-0.209	** 0.001	0.066	0.120
PuM	-2.936	0.003	** 0.008	-0.241	*** <0.001	-0.008	0.879
LGN	-1.160	0.246	0.246	N/A	N/A	N/A	N/A
PuI	-1.289	0.198	0.231	N/A	N/A	N/A	N/A

Supplementary table 3: **Statistical summary from the interaction analysis between the regions of interest and APOE4 in predicting performance on the immediate recall test using robust linear regression.** The Spearman's ρ and the p values are represented for interactions surviving FDR correction. * p<0.05, ** p <0.01, *** p <0.001. ROI = region of interest, APOE4 = apolipoprotein ε4, FDR = false discovery rate, MGN = Medial geniculate, LSG = Limitans suprageniculate, PuA = Pulvinar anterior, PuL = Pulvinar lateral, PuM = Pulvinar medial, LGN = Lateral geniculate, PuI = Pulvinar inferior.
